# A *Vavraia*-like microsporidium as the cause of deadly infection in threatened and endangered *Eurycea* salamanders in the United States

**DOI:** 10.1186/s13071-019-3369-z

**Published:** 2019-03-14

**Authors:** Xue Yu, Rachel L. Hoyle, Fengguang Guo, Cameron M. Ratliff, Valentin Cantu, Justin Crow, Lixin Xiang, J. Jill Heatley, Guan Zhu

**Affiliations:** 1 Department of Veterinary Pathobiology, College of Veterinary Medicine & Biomedical Sciences, Texas A&M University, College Station, Texas USA; 2 Department of Veterinary Small Animal Clinical Sciences, College of Veterinary Medicine and Biomedical Sciences, College Station, Texas USA; 3 United States Fish and Wildlife Service, San Marcos Aquatic Resources Center, San Marcos, Texas USA; 4 College of Life Sciences, Zhejiang University, Hangzhou, Zhejiang China

**Keywords:** Lungless salamanders, *Eurycea sosorum*, *Eurycea nana*, Endangered species, Microsporidiosis, *Vavraia*-like microsporidian, Aquatic invertebrates, Reservoir

## Abstract

**Background:**

*Eurycea sosorum* (Barton Springs salamander) and *Eurycea nana* (San Macros salamander) are listed as endangered and threatened species, respectively, by the U.S. Fish and Wildlife Service (USFWS) with habitats restricted to small regions near Austin, Texas, USA. The conservation efforts with the *Eurycea* salamanders at the captive breeding program in San Marcos Aquatic Resources Center (SMARC), a USFWS facility, have seen an unexpected and increased mortality rate over the past few years. The clinical signs of sick or dead salamanders included erythema, tail loss, asymmetric gills or brachial loss, rhabdomyolysis, kyphosis, and behavior changes, suggesting that an infectious disease might be the culprit. This study aimed to identify the cause of the infection, determine the taxonomic position of the pathogen, and investigate the potential reservoirs of the pathogen in the environment.

**Results:**

Histopathological examination indicated microsporidian infection (microsporidiosis) in the sick and dead *Eurycea* salamanders that was later confirmed by PCR detection. We also determined the near full-length small subunit ribosomal RNA (*SSU* rRNA) gene from the microsporidian pathogen, which allowed us to determine its phylogenetic position, and to design primers for specific and sensitive detection of the pathogen. Phylogenetic analysis indicated that this pathogen was closely related to the insect parasites *Vavraia* spp. and the human opportunistic pathogen, *Trachipleistophora hominis*. This *Vavraia*-like microsporidium was present in dead salamanders at SMARC archived between 2011 and 2015 (positive rates ranging between 52.0–88.9% by PCR detection), as well as in some aquatic invertebrates at the facility (e.g. snails and small crustaceans).

**Conclusions:**

A *Vavraia*-like microsporidian was at least one of the major pathogens, if not solely, responsible for the sickness and mortality in the SMARC salamanders, and the pathogen had been present in the center for years. Environmental invertebrates likely served as a source and reservoir of the microsporidian pathogen. These observations provide new knowledge and a foundation for future conservation efforts for *Eurycea* salamanders including molecular surveys, monitoring of the pathogen, and discovery of effective treatments.

**Electronic supplementary material:**

The online version of this article (10.1186/s13071-019-3369-z) contains supplementary material, which is available to authorized users.

## Background

**Fig. 1:**
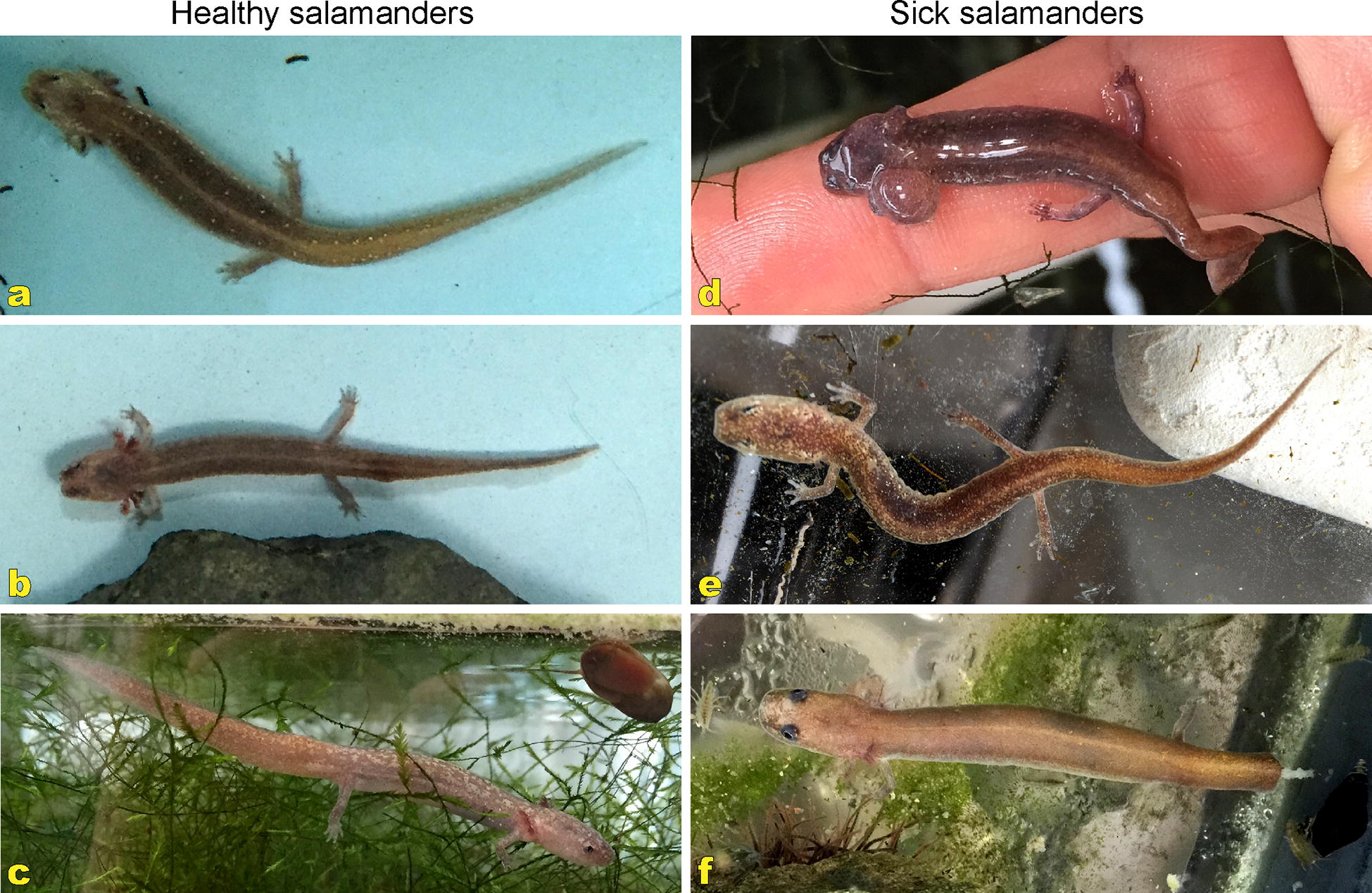
Healthy and sick *Eurycea* salamanders. Photographs were taken at the San Marcos Aquatic Resource Center (SMARC), Texas, to compare the appearances of healthy (**a**-**c**) and sick (**d**-**f**) *Eurycea* salamanders to illustrate the impact of *Vavraia*-like microsporidian infection on the animals

The Barton Springs salamander *Eurycea sosorum* and the San Macros salamander *Eurycea nana* are listed as endangered and threatened species, respectively (Fig. 1) [1, 2]. These two species are small lungless salamanders (family Plethodontidae) with an adult length up to ~7.5 cm for *E. sosorum* and 8–9 cm for *E. nana.* The habitat of *E. sosorum* is exclusive to Barton Springs in Austin, and the habitat of *E. nana* encompasses Spring Lake and a small region of the headwater of the San Marcos River near Austin, Texas, USA [3, 4]. The San Marcos Aquatic Resources Center (SMARC) is a United States Fish and Wildlife Service (USFWS) facility located on the south side of Austin with the primary mission “to provide support for, and undertake research on, endangered, threatened, and species at risk” (https://www.fws.gov/southwest/fisheries/san_marcos/index.html), including the efforts on *E. sosorum* and *E. nana*. In the past decade, there has been a progressive increase in salamander mortality at SMARC, where up to a 10% fatality rate was observed during the summer months in some tanks hosting *E. sosorum* and *E. nana*, which threatened the conservation of these two salamander species.

Here we present histological and molecular evidence that microsporidiosis was a major cause (if not the only cause) of the observed increase in sickness and death of these two lungless salamander species at SMARC. We have cloned and sequenced the small subunit ribosomal RNA (*SSU* rRNA) gene from the microsporidian parasite, and determined that it was closely related to the insect parasites *Vavraia* spp. and the opportunistic pathogen *Trachipleistophora hominis*. While more comprehensive morphological and molecular data are needed to name the exact species of the parasitic microsporidium, we were able to develop a nested PCR-based method to detect the presence of the pathogen in the salamanders for epidemiological surveys and in the zooplanktons for identification of potential sources of the pathogen.

## Methods

### Salamander specimens and collection of their food sources

Six *E. sosorum* salamanders, collected shortly after death, were sent to the Zoological Medicine Service, Department of Veterinary Small Animal Clinical Sciences and the Zhu Lab in the Department of Veterinary Pathobiology at Texas A&M University from SMARC in the summer of 2013 for diagnosis including necropsy and histological examinations. Additional archived corpses preserved in 70% ethanol were obtained from SMARC for isolating DNA for a molecular epidemiological survey.

In order to identify potential sources of the microsporidian parasite, invertebrates representing food sources and environmental samples were collected at SMARC. Two species of hatchery-raised snails were collected from the circular snail tank (*Helisoma anceps*) or refugia tanks (*Elimia comalensi*), black worms (*Lumbriculus variegatus*) were collected from the refugia tanks, brine shrimps (*Artemia salina*) were collected from a tank manufactured by Mariculture Technologies International (Oak Hill, FL, USA) and used for storing live brine shrimp, amphipods (*Hyalella azteca*) were collected from a water raceway, and other zooplanktons, including water fleas, seed shrimps, backswimmers, calinoids and cyclopods, were obtained from hatchery ponds B6 and D8 several times using a fine mesh net. The refugia tanks were used to host sick salamanders and were where dead salamanders were found. All invertebrates and environmental samples (*n* ≥ 12 for each species) were individually identified and collected under a dissecting microscope, kept alive in Ziploc plastic bags filled with water, and transported from SMARC to the laboratory in the Department of Veterinary Pathobiology, Texas A&M University on the same day in a cooler and stored at 4 °C in the refrigerator overnight before extracting DNA. Fish food flakes purchased from Pentair Aquatic Eco-systems (Apopka, FL, USA) and used to feed salamanders at SMARC were also collected.

### Isolation of DNA

Total DNA samples were isolated from two types of dead salamanders from SMARC. The first types of samples were the corpses of fresh frozen *E. sosorum* salamanders sent to the Zoological Medicine Services, Small Animal Hospital, Texas A&M University for diagnosis in 2013 (*n* = 6). DNA samples were isolated from frozen tissues containing lesions after necropsy and histological examinations were performed using DNeasy Blood & Tissue kits (Qiagen, Valencia, CA, USA) following the manufacturer’s standard protocol. The second type of samples were the ethanol-fixed salamander corpses collected between 2011 and 2015. These archived specimens contained both *E. sosorum* and *E. nana* species.

Among the archived specimens, tissues from the tail, gut and gill were collected from each corpse and combined into a 1.5 ml microtube, washed three times using phosphate-buffered saline (PBS, pH7.4) and subsequently centrifuged. Each DNA extraction used up to 25 mg of the combined tissues using the Qiagen DNeasy Blood & Tissue kit following the manufacturer’s instruction with slight modifications. Briefly, 180 µl of ATL buffer solution was added into the tube, followed by three freeze/thaw cycles. The samples were then incubated at 95 °C for 20 min and allowed to cool down to room temperature, followed by the addition of 20 µl of proteinase K (20 mg/ml) and incubation at 56 °C for 3 h, with vortexing every 15 min until the solution became fully homogenized. The remaining procedures followed the manufacturer’s instruction for the Qiagen DNeasy Blood & Tissue kit.

For isolating DNA from aquatic invertebrates, snails were individually minced with razor blades, while zooplanktons were collected by centrifugation at 4000×*g* for 10 min. All zooplanktons of the same species were mixed together as one specimen in 1.5 ml Eppendorf tubes. Up to 25 mg of each specimen was collected and ground in a small plastic pestle containing 180 µl of ATL buffer solution, followed by three freeze/thaw cycles. The remaining procedures followed those for extracting DNA from salamander corpses as described above [5, 6]. All DNA samples were eluted twice, each with 15 µl of AE elution buffer solution, and stored at  - 20 °C until use.

### Detection of microsporidian *SSU* rRNA

After strong clinical and histopathological indication of microsporidian infection in the *Eurycea* salamanders, we first attempted to amplify a fragment of *SSU* rRNA from the frozen tissues of the six fresh salamanders sent to the Small Animal Hospital by nested PCR using protocol and primers previously described for detecting microsporidia in fecal samples from wild quail [7]. Universal primers used in this experiment included Microspor_270F and Microspor_840R in primary PCR, and Microspor_446F and Microspor_776R in secondary PCR (Additional file 1: Table S1). PCR products (473 bp) were purified using an E.Z.N.A Cycle Pure Kit (Omega Biotek, Norcross, GA, USA), according to the manufacturer’s protocol, for automated Sanger sequencing using universal primers (both directions).

Based on the 473-bp microsporidian sequence obtained using universal primers, specific primers were designed for more specific and sensitive detection of the microsporidium in archived salamander corpses and other specimens (i.e. Microsp_Salam_S1F and Microsp_Salam_S1R for primary PCR, and Microsp_Salam_S2F and Microsp_Salam_S2R for secondary PCR) (see a list of primers used in this study in Additional file 1: Table S1).

In a typical nested PCR detection of microsporidium in archived salamanders and other specimens, the primary PCR reaction (20 µl/well) contained 2.0 µl of DNA, 2.0 µl of 1.0 µM each of the primers specific for the microsporidium infecting the salamander (Microsp_Salam_S1F and Microsp_Salam_S1R), 5.0 µl of nuclease-free water, 1.0 µl of DMSO and 10 µl of Red-Taq ReadyMix PCR reaction mix (Sigma-Aldrich, St. Louis, MO, USA). Reactions started with a denaturation step at 95 °C for 5 min, followed by 10 thermal cycles at 95 °C for 45 s, 60 °C for 45 s and 72 °C for 60 s, and 35 cycles at 95 °C for 30 s, 55 °C for 30 s and 72 °C for 45 s, with an extension at 72 °C for 5 min after all the reactions. The secondary PCR reaction (20 µl/well) contained 0.5 µl of the final product from the primary PCR reaction, 2.0 µl of 1.0 µM each of the primers specific for the salamander microsporidia (Microsp_Salam_S2F and Microsp_Salam_S2R), 6.5 µl of nuclease-free water, 1.0 µl of DMSO and 10 µl of Red-Taq ReadyMix PCR reaction mix. The secondary PCR reactions started with a denaturation step at 95 °C for 5 min, followed by 20 thermal cycles at 95 °C for 30 s, 55 °C for 30 s and 72 °C for 45 s, with an extension at 72 °C for 5 min after all the reactions.

PCR reactions were performed on a T100 Thermal Cycler (Bio-Rad Laboratories, Hercules, CA, USA), and the PCR products were fractionated with 1.5% agarose gel mixed with ethidium bromide and visualized in a Hitachi Genetic Miraibio CCDBIO Imaging System (MiraiBio, Alameda, CA, USA). Each set of reactions contained a reagent negative control using nuclease-free water as a template, a sample negative, and a positive control using DNA isolated from tissues of microsporidian-negative and positive salamanders, respectively. Experiments were repeated if the controls failed to show expected results. All the microsporidia detections were repeated at least twice with consistent results. A selected number of PCR products were sequenced and analyzed to verify their identity.

### Determination of the near full-length microsporidian *SSU* rRNA and phylogenetic reconstruction

In order to gain more information on the taxonomic position of the microsporidium infecting *Eurycea* salamanders, we designed new universal primers based on *Vavraia* and related species that were used with specific primers located within the first piece of the 473-bp *SSU* rRNA sequence for amplifying the near full-length microsporidian *SSU* rRNA gene by overlapping PCR. The primer pairs included Microsp_univ01F and Microsp_Salam_S1R for determining 5′ end sequences, and Microsp_Salam_S2F and Microsp_univ02R_1336_19 for determining 3′ end sequences (Additional file 1: Table S1). PCR templates used DNA samples isolated from the frozen tissues collected in 2013. PCR amplicons were purified and used for sequencing. Overlapping sequences were assembled using MacVector v.15.0 or higher. The obtained near full length *SSU* rRNA gene sequence was deposited in the GenBank database under the accession number MH918740.

For sequence analysis, the near full-length microsporidian *SSU* rRNA sequence (1337 bp) was used as a query to search orthologs in the non-redundant nucleotide databases at the National Center for Biotechnology Information (NCBI) using BLASTN (http://www.ncbi.nlm.nih.gov). Up to 1000 highly homologous sequences were retrieved, which were subjected to multiple sequence alignments (MSAs) with the sequence obtained in this study using a Unix version of the MUSCLE program (v.3.8.31) [8]. After the alignment, partial sequences, nearly identical sequences, and those containing multiple ambiguous nucleotides were removed. The retained sequences were aligned again and subjected to a “rough” phylogenetic reconstruction by neighbor-joining (NJ) method based on Tamura-Nei distance [9–11]. Based on the rough NJ tree, 71 sequences (dataset 1) representing major taxonomic groups were retained and subjected to MSAs. The aligned sequences were visually inspected to correct apparent errors, and all gaps and questionable positions were removed.

Phylogenetic trees were inferred from the trimmed dataset by Bayesian inference (BI) method using MrBayes (v.3.2) (http://mrbayes.sourceforge.net) [12], in which one million generation of searches were performed with four independent chains running. The BI analysis used a general time reversal (GTR) nucleotide substitution model with the inclusion of fraction of invariance and 16-rate of discrete gamma (GTR + F_inv_ + Γ_16_) considerations. Searches converged as determined by the average standard deviation of split frequencies reaching to < 0.01, and potential scale reduction factor (PSRF) values for variance approaching 1.0 [13].

Based on the BI tree inferred from the 71-taxa (dataset 1), a second dataset containing 37 sequences that were closely related to the sequence in this study was similarly built using the programs MSA with MUSLE, which resulted in more positions being aligned (dataset 2). Dataset 2 was subjected to phylogenetic reconstructions by BI and maximum likelihood (ML) methods. The BI analysis used the same approaches as described for the dataset 1. The ML analysis was performed using the program MEGA (v.6.0) (https://www.megasoftware.net/) [14], in which 1000 ML tress were obtained by bootstrapping analysis using GTR + *F*_inv_ + *Γ*_16_ parameters. In all analyses, the majority rule consensus trees were displayed using the program FigTree (v.1.4.2) (http://tree.bio.ed.ac.uk/software/figtree/), followed by illustration using Adobe Illustrator CC (http://www.adobe.com).

## Results

### Histopathological indication of microsporidian infection in salamanders

**Fig. 2:**
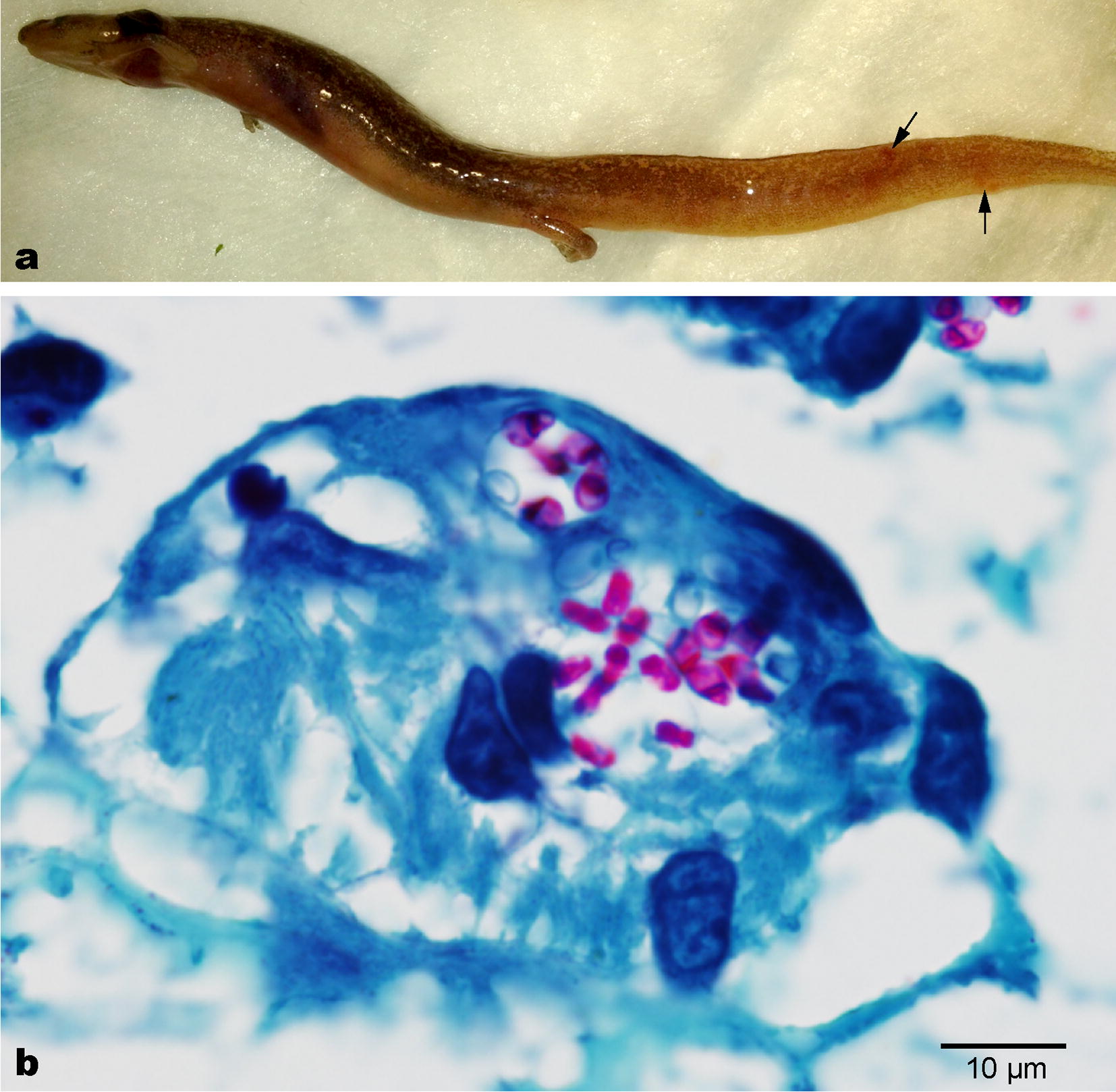
Histopathological examination. **a** Appearance of a dead *Eurycea sosorum* salamander submitted to the Zoological Medicine Service for sacrifice and necropsy. Representative red pinpoint lesions in a sick *Eurycea sosorum* salamander are indicated by arrows. **b** Histopathological detection of microsporidia cysts (stained in red) in a representative microscopic section from a lesion by Giemsa staining (Photo courtesy Drury Reavill, Zoo/Exotic Pathology Service)

Six *E. sosorum* salamanders were sent to the Texas A&M University Small Animal Hospital from SMARC shortly after death for diagnosis in the summer of 2013. These specimens (as well as other sick salamanders at SMARC) displayed various clinical signs including abnormal light or dark spots on the skin, digit or distal tail loss, and/or asymmetrical gill or branchial arch loss (Fig. 1; Additional file 1: Table S2). Mild to severe red pinpoint lesions were also visible under the skin of some sick or dead salamanders (Fig. 2a). Histopathology revealed lymphocytic and granulocytic inflammation of muscle throughout the bodies and tails of multiple salamanders and identified as focally extensive subacute rhabodomyositis. No associated microbe was identified; however, in areas of some animals, histiocytic cells and/or cysts morphologically suggestive of a possible microsporidia parasite were also identified. The diagnosis of microsporidiosis was supported by Giemsa-stained histopathological examination, in which intracellular pathogens characteristic of microsporidia were observed in red (Fig. 2).

### A *Vavraia*-like microsporidium in the family Pleistophoridae was the causative agent of the microsporidiosis in *E. sosorum* and *E. nana* salamanders

**Fig. 3:**
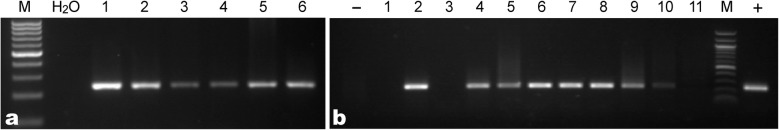
Nested PCR detection of *Vavraia*-like microsporidian DNA from *Eurycea* salamanders from SMARC. **a** Agarose gel image of PCR products derived from the frozen tissues of the six salamander specimens collected in 2013 outbreak for initial diagnosis and molecular detection. Negative control (−) used pure water. **b** Agarose gel image of PCR products derived from a selected number of archived salamander specimens indicating both negative and positive detection. Negative (−) and positive (+) controls used DNA isolated from microsporidian-negative and -positive salamander specimens. Nested PCR reactions used primer pairs Microsp_Salam_S1F and Microsp_Salam_S1R for primary PCR, and Microsp_Salam_S2F and Microsp_Salam_S2R for secondary PCR as described in Additional file 1: Table S1

The histopathological diagnosis was further confirmed by PCR detection of microsporidian *SSU* rRNA gene from the six salamanders. Because of the lack of knowledge on the specific microsporidian species infecting *Eurycea* salamanders, we initially employed nested PCR using universal primers previously used to detect microsporidia in wild quail [7]. We were initially able to produce amplicons from three of the six salamanders due to the imperfect homology of these primers with the target sequence. Sequencing confirmed the identity of the 473-bp PCR products as a fragment of microsporidian *SSU* rRNA gene, in which the top hits in the NCBI databases were the *SSU* rRNA gene sequences from various species of Pleistophoridae microsporidia (e.g. *Trachipleistophora* sp., *Vavraia culicis* and *Microsporidium* sp., all with identity = 95% and an E-value of 0.0). The availability of the sequence allowed us to design specific primers for more sensitive detection that confirmed microsporidian infection in all six salamanders (Fig. 3a; Additional file 1: Table S2).

To gain a better knowledge of the microsporidian pathogen, we amplified and determined 1337 bp sequences covering the majority of the *SSU* rRNA gene with the top hits from *Vavraia culicis*, *Trachipleistophora hominis* and related species (highest identity = 96%; E-value = 0). However, we were unable to determine the exact species based on the *SSU* rRNA gene sequence due to the lack of a 100% match or close to 100% match in the databases.

**Fig. 4:**
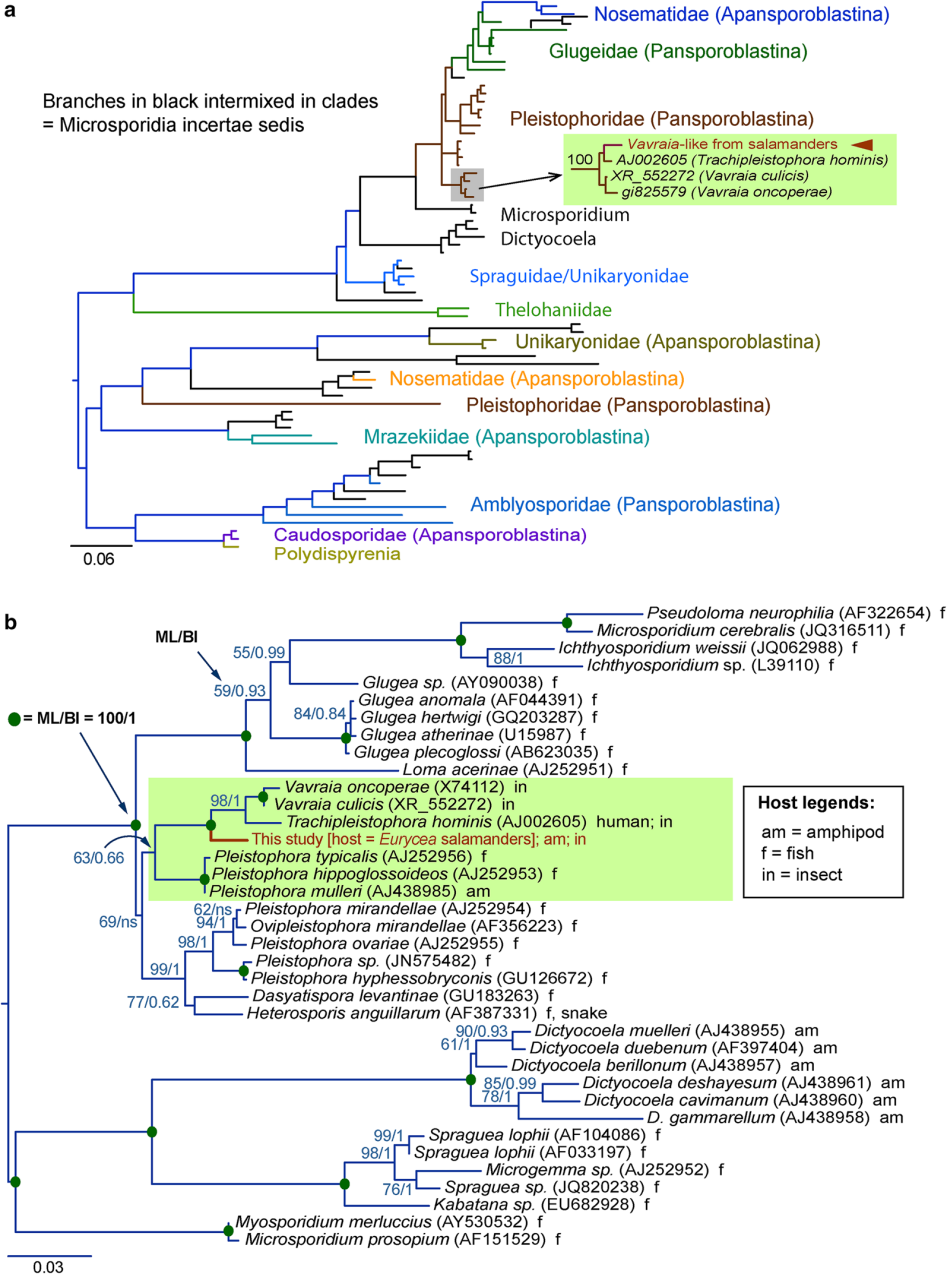
Phylogenetic analysis of the *Vavraia*-like microsporidium based on the near full-length *SSU* rRNA genes. **a** Phylogenetic tree reconstructed by the Bayesian inference (BI) method based on the large dataset (dataset 1 as described in Methods) to determine the phylogenetic position of the *Vavraia*-like microsporidium among various microsporidian taxonomic groups. **b** Phylogenetic tree reconstructed by BI and maximum likelihood (ML) methods based on a small dataset (dataset 2 as described in Methods) to fine-tune the phylogenetic position of the *Vavraia*-like microsporidium. Posterior probability values in BI analysis and bootstrap supporting values in ML analysis are indicated at the nodes. Solid circles at the nodes indicate 100% support by both analyses

Phylogenetic reconstructions also placed the microsporidian sequence obtained in this study within the clade containing species from the family Pleistophoridae that was 100% supported by posterior probability value (Fig. 4a). The *SSU* rRNA sequence obtained in this study was placed at the base of the clade consisting of insect parasite species *Vavraia* and opportunistic pathogen *Trachipleistophora hominis* with 100% support by posterior probability value (Fig. 4a). A more detailed analysis with taxa from the family Pleistophoridae and taxonomically related species also placed the microsporidian sequence obtained in this study at the base of the clade consisting of insect parasite species *Vavraia* and opportunistic pathogen *T. hominis* [15], which was again reinforced by posterior probability and bootstrap supporting values (Fig. 4b).

Based on the results of BLASTN searches and phylogenetic analysis, we have molecularly confirmed that the two *Eurycea* salamanders at SMARC were infected by a *Vavraia*-like microsporidium. A more comprehensive morphological and molecular analyses are needed to determine whether this *Vavraia*-like pathogen is a new species or a previously described species without molecular sequence data in GenBank (accession number: MH918740).

### Microsporidiosis appeared to be a major cause of death in the archived salamanders

Because the clinical signs associated with microsporidiosis had been observed in sick and dead *Eurycea* salamanders at SMARC for years, we decided to conduct a molecular epidemiological survey on the ethanol-fixed dead salamanders that were collected at SMARC between 2011 and 2015 (*n *= 93) by nested PCR using specific primers designated for detection (Additional file 1: Table S1). Among the archived salamanders, we detected *Vavraia*-like microsporidium in specimens collected in all five years (Table 1; Fig. 3b). When the six specimens sent to TAMU for diagnosis in 2013 were also included for the purpose of unbiased statistical analysis, the positive rates ranged from 52.0% in 2013 to 88.9% in 2011, and the overall positive rate of all samples was 65.3%. While the data was only applicable to salamanders that died for various reasons, rather than live animals, the observation indicated that the *Vavraia*-like microsporidian has been present at SMARC for years.

**Table 1:** The positive rates of *Vavraia*-like microsporidium in archived salamanders collected between 2011 and 2015 as detected by nested PCR

Year	Total (*n*)	Positive (*n*)	Positive rate (%)
2011	9	8	88.9
2012	12	10	83.3
2013^a^	25	13	52.0
2014	15	9	60.0
2015	38	24	63.2
Total	98	64	65.3

^a^Data for 2013 included the 6 specimens sent to the Texas A&M University for diagnosis for the purpose of unbiased statistical analysis

### Some aquatic invertebrates were the potential sources of the microsporidian parasite infecting salamanders

Because the native hosts for *Vavraia* species are insects [16–23], and the opportunistic *Trachipleistophora* species as well [24–29], we suspected that the *Vavraia*-like pathogen afflicting salamanders at SMARC was also a parasite native to insects, but capable of causing “opportunistic infections” in salamanders under the unnatural environment at SMARC. We hence performed a molecular detection for the *Vavraia*-like microspordium in the food sources or co-existing species in the aquatic environment at SMARC including fish food flakes and selected invertebrates.

Among the 11 types of samples obtained from the ponds at SMARC, we detected the presence of *Vavraia*-like microsporidian parasite in the two species of snails (i.e. *H. anceps* and *E. comalensi*) and in the cyclopods including two types of crustaceans, i.e. water fleas (order Cladocera) and cyclopods (order Cyclopoida) (Additional file 1: Table S3). Together with the fact that *Vavraia* species are native parasites of insects, these observations strongly suggest that zooplanktons are likely the native hosts and sources of the *Vavraia*-like parasite afflicting the *Eurycea* salamanders in SMARC, with some snails possibly serving as reservoirs.

Additionally, this microsporidium was also detectable by nested PCR in some of the tank sediments where sick or dead salamanders were found, which suggests a low level of contamination of microsporidia in the water circulation system in the facility during the outbreaks. *Eurycea* salamanders were hosted in a temperature and light controlled facility, which excludes other major environmental and biological factors that may cause the death of *Eurycea* salamanders. The quality of water in the facility was consistently monitored, and a test during an outbreak showed the water quality met the following criteria: temperature = 74.5 °F (23.6 °C), pH = 7.6, NH_3_ = 0 ppm, NH_4_^+^ = 0 ppm, NO_2_ = 0 ppm and Cl^−^ = 0 ppm. In salamanders, some levels of manifestation of gastrointestinal parasitism (e.g. nematodes, cestodes, and occasional pyriform protozoa resembling *Giardia*) were found in both healthy and sick salamanders, but there was no apparent association between these parasites and increased mortality. On the other hand, the increased mortality was mostly associated with the deformation of salamanders (e.g. skin lesions, loss of tails and digit), in which only a microsporidia-like organism was observed.

## Discussion

Microsporidia are a group of unicellular and obligate intracellular parasites, which are closely related to fungi [30]. Microsporidia are known as important opportunistic parasites of immunocompromised patients and infect many animals including amphibians, fishes and reptiles [31–33]. A few cases of infectious diseases in salamanders have been reported, including one reported microsporidia infection [34–37].

We discovered that salamanders in SMARC were infected with *Vavraia*-like microsporidia and that those microsporidia also existed in some aquatic invertebrates, which served as food sources for the salamanders or were present in their habitat. Since the native hosts of *Vavraia*-like microsporidium are insects, it is highly likely that the aquatic invertebrates served as reservoirs for the microsporidia. After contact between aquatic invertebrates and salamanders through feeding and interactions, the *Vavraia*-like microsporidium caused opportunistic infection in *E. sosorum* and *E. nana* salamanders due to stress in an unnatural environment.

Based on these discoveries, SMARC staff have stopped feeding salamanders with contaminated invertebrates and removed the two species of snails from the tanks to avoid the transmission of *Vavraia*-like microsporidium from zooplanktons or snails to the salamanders. In doing so, they have observed certain levels of reduced mortality of salamanders (based on personal communications). However, the practice could not fully eliminate the sickness of salamanders in SMARC, which is likely due to the presence of *Vavraia*-like microsporidium in the natural environment, such as in the zooplanktons in the water system. Because of this, it was advised to quarantine the newly captured wild salamanders from those born in the center to reduce the potential spread of infection. When resources permit, a water filtration system might be installed to reduce the introduction of zooplanktons from the environmental water to SMARC.

On the other hand, it is truly difficult or impossible to completely eliminate the microsporidia-carrying zooplanktons in SMARC or in the field. Historically, microsporidian *Steinhausia* sp. is known to be the cause of extinction of a land snail *Partula turgida* from French Polynesia [38]. Rapid evolution of virulence might also lead to host extinction under host-parasite coevolution as demonstrated by the coevolution experiment using the red flour beetle, *Tribolium castaneum*, and its natural microsporidian parasite, *Paranosema whitei* [39]. Therefore, it may be necessary to discover effective therapeutics to treat the *Vavraia*-like microsporidian infection in *E. sosorum* and *E. nana* before the pathogen evolves to be more virulent and deadly to the shrinking population of endangered *Eurycea* salamanders.

The close-relationship of the *Vavraia*-like microsporidium with the known opportunistic pathogen *T. hominis* also raised a concern about the potential of this *Vavraia*-like microsporidium to infect other vertebrates, including humans. It could be a water-borne and vector-borne threat to public health, considering that most *V. culicis* infections were reported in mosquitoes with larval stages in water [17, 22, 40]. For this concern, the SMARC center was advised to warn individuals with a compromised or weakened immunity such as patients with acquired immune deficiency syndrome (AIDS), infants and the elderly to avoid direct contact with the animals and water system in the center.

In the present study, we also developed a nested PCR method for the sensitive and specific detection of the *Vavraia*-like microsprodium. This method could be applied to the diagnosis, detection and survey of the *Vavraia*-like parasite in salamanders raised in SMARC and in the field, as well as pathogens in other aquatic species.

## Conclusions

We have provided histopathological and molecular evidence that a *Vavraia*-like microsporidian was at least one of the major pathogens, if not solely, responsible for the sickness and mortality of two lungless *Eurycea* salamanders in Texas, United States. Some environmental invertebrates likely served as a source and reservoir of the microsporidian pathogen. We also developed a nested PCR protocol for diagnosis, detection and survey of the *Vavraia*-like pathogen. This study provides new knowledge and a foundation for future conservation efforts for *Eurycea* salamanders including molecular surveys, monitoring of the pathogen, and discovery of effective treatments.

## Additional file

**Additional file 1: Table S1.** Description of primers used in this study. **Table S2.** Comparison between clinical descriptions and PCR detection of microsporidia of the six salamanders from the 2013 outbreak. **Table S3.** The detection of microsporida in salamander food sources and inveterbrates in the environment using primers developed in this study.

## Abbreviations

AIDS: acquired immune deficiency syndrome
BI: Bayesian inference
GTR: general time reversal
ML: maximum likelihood
NCBI: National Center for Biotechnology Information
NJ: neighbor-joining
PBS: phosphate-buffered saline
PCR: polymerase chain reaction
PSRF: potential scale reduction factor
SMARC: San Marcos Aquatic Resource Center
*SSU* rRNA: small subunit ribosomal RNA
USFWS: United States Fish and Wildlife Service
