# Supplementary material for: A Vavraia-like microsporidium as the cause of deadly infection in threatened and endangered Eurycea salamanders in the United States
Source: Parasit Vectors. 2019 Mar 14;12:108. doi: 10.1186/s13071-019-3369-z (PMC6419446; doi:10.1186/s13071-019-3369-z)
Supplement: Supplementary file 1 — Additional file 1: Table S1. Description of primers used in this study. Table S2. Comparison between clinical descriptions and PCR detection of microsporidia of the six salamanders from the 2013 outbreak. Table S3. The detection of microsporida in salamander food sources and inveterbrates in the environment using primers developed in this study. [file 13071_2019_3369_MOESM1_ESM.docx]

**Additional file 1: Table S1**. Description of primers used in this study

| **Primer name** | **Primer sequence (5′ to 3′)** | **Length (nt)** | **Position in SSU rRNA^†^** | **Application** |
| --- | --- | --- | --- | --- |
| Microspor_270F^‡^ | GCCTGAGATGGCTMYACG | 21 | 297 | Universal primers for Primary PCR |
| Microspor_840R^‡^ | GTTGAGTYAAATTAAGCMGCACA | 23 | 884 |  |
| Microspor_446F^‡^ | GRATGCTGCAGTTAAARDGTC | 21 | 477 | Universal primers for Secondary PCR |
| Microspor_776R^‡^ | TCACYCTTGCGAGCRTACTAT | 21 | 819 |  |
| Microsp_univ01F | CACCAGGTTGATTCTGCCTGAC | 21 | 1 | Amplification of long SSU rRNA |
| Microsp_univ02R | GYTRCCTTGTTACGACTT | 18 | 1337 |  |
| Microsp_Salam_S1F | GTACGATGTGCAGGTAAAGTA | 21 | 385 | Specific primers for detection; Primary PCR |
| Microsp_Salam_S1R | ACGTCCTTTATCATCGGAATC | 21 | 683 |  |
| Microsp_Salam_S2F | ACGATGTGCAGGTAAAGTATG | 21 | 387 | Specific primers for detection; Secondary PCR |
| Microsp_Salam_S2R | CGAGTCATCAAATTTCACCTC | 21 | 625 |  |

^†^ Position refers to the position of the primer’s 5′ end in the SSU rRNA gene sequence of the *Vavraia*-like parasite determined by this study (GenBank: MH918740).

^‡^ Adapted from [7]. The number within the primer name indicates the position of the primer’s 5′ end in the reference sequence XR_002670150 (SSU rRNA gene from *Encephalitozoon intestinalis* ATCC 50506).

**Additional file 1: Table S2.** Comparison between clinical descriptions and PCR detection of microsporidia of the six salamanders from the 2013 outbreak

| Salamander specimens | Clinical implication and histopathology | PCR detection of microsporidia | |
| --- | --- | --- | --- |
|  |  | Universal primers | Specific primers |
| S1 | Mild rhabdomyositis | + | + |
| S2 | Subacute rhabdomyositis | + | + |
| S3 | Intestinal cestodiasis, ovarian folliculitis, multifocal rhabdomyositis and steatitis | _ | + |
| S4 | Cestodiasis | _ | + |
| S5 | Moderate lymphocytic rhabdomyositis | _ | + |
| S6 | Rhabomyositis, intralesional *microsporidia* | + | + |

**Additional file 1:** **Table S3.** The detection of microsporida in salamander food sources and inveterbrates in the environment using primers developed in this study.

| **Species** | **Original location on the SMARC** | | **PCR Detection** |
| --- | --- | --- | --- |
| Snails *(Helisoma anceps )* | QW-5: A flow-through tank system for salamanders | | + |
| Snails *(Elimia comalensis)* | Circular snail tank | | + |
| Amphipoda *(Hyalella azteca)* | Water raceways 1 and 2 | | - |
| Black worms *(Lumibriculus variegatus)* | EN4-4/QW-7 : A flow-through tank system for salamanders | | - |
| Brine shrimp *(Artemia salina)* | BSB Brine Shrimp Tank | | - |
| Fish food flakes | Freezers | | - |
| Water fleas (Cladocera) | Ponds D8/B6: | Two separate ponds to raise invertebrates to feed salamanders | +/- |
| Cyclopods (Cyclopoida) | Ponds D8/B6 |  | -/+ |
| Seed shrimps (Ostracod) | Ponds D8/B6 |  | -/- |
| Copepods (Calanoida) | Ponds D8/B6 |  | -/- |
| Back swimmer (Notonectidae) | Ponds D8/B6 |  | -/- |
